# A pain relieving reimbursement program? Effects of a value-based reimbursement program on patient reported outcome measures

**DOI:** 10.1186/s12913-020-05578-8

**Published:** 2020-08-27

**Authors:** Thérèse Eriksson, Hans Tropp, Ann-Britt Wiréhn, Lars-Åke Levin

**Affiliations:** 1 Department of Health, Medicine and Caring Sciences (HMV), Centre for Medical Technology Assessment (CMT), Linköping University, SE-581 83 Linköping, Sweden; 2 Department of Biomedical and Clinical Sciences, Linköping University, SE-581 83 Linköping, Sweden; 3 Research and Development Unit in Region Östergötland and Department of Medical and Health Sciences, Linköping University, SE-581 83 Linköping, Sweden

**Keywords:** Reimbursement, Payment, Value-based, Bundled payment, P4P, Incentives, PROM, ODI, EQ-5D

## Abstract

**Background:**

Value**-**based reimbursement programs have become increasingly common. However, little is known about the effect of such programs on patient reported outcomes**.** Thus, the aim of this study was to analyze the effect of introducing a value-based reimbursement program on patient reported outcome measures and to explore whether a selection bias towards less complicated patients occurred.

**Methods:**

This is a retrospective observational study with a before and after design based on the introduction of a value-based reimbursement program in Region Stockholm, Sweden. We analyzed patient level data from inpatient and outpatient care of patients undergoing lumbar spine surgery during 2006–2015. Patient reported outcome measures used was Global Assessment, EQ-5D-3L and Oswestry Disability Index. The case-mix of surgically treated patients was analyzed using medical and socioeconomic factors.

**Results:**

The value-based reimbursement program did not have any effect on targeted or non-targeted patient reported outcome measures. Moreover, the share of surgically treated patients with risk factors such as having comorbidities and being born outside of Europe increased after the introduction. Hence, the value-based reimbursement program did not encourage discrimination against sicker patients. However, the income was higher among patients surgically treated after the introduction of the value-based reimbursement. This indicates that a value-based reimbursement program may contribute to increased inequalities in access to healthcare.

**Conclusions:**

The value-based reimbursement program did not have any effect on patient reported outcome measures. Our study contributes to the understanding of the effects of a value-based reimbursement program on patient reported outcome measures and to what extent cherry-picking arises.

## Background

Governance within healthcare is complex due to information asymmetry caused by the inherent agency connections between stakeholders with different objectives [1]. Reimbursement programs seek to align these objectives through financial incentives [2] but too strong or too weak incentives are often accompanied with unintended consequences [3, 4]. To better align financial incentives with professional values, a value-based reimbursement program (VBRP) combines different payment models. In theory, a VBRP entail both quality enhancing and cost-containing incentives to generate value [5].

Surgical procedures is considered suitable for VBRP given the distinct beginning and end of a care episode. Spine surgery is considered particularly suitable, since the appropriateness of surgery compared to conservative treatment among patients with low back pain is debated and recommendations in clinical guidelines vary [6]. Thus, highlighting the importance of preventing patient selection based on medical irrelevant factors, such as socioeconomic status. Moreover, since low back pain is estimated to affect 80–85% of the world’s population [7] with a large and growing economic burden [8], a well-functioning reimbursement program within spine surgery is important.

In this study, we analyze the introduction of a VBRP within elective spine surgery in Region Stockholm, Sweden. The Stockholm VBRP (STHLM-VBRP) combines bundled payment with pay-for-performance (P4P). The bundled payment extends the clinical episode to 1 year after surgery, which is a longer period compared to most other bundled payment programs previously assessed [9, 10]. The P4P is based upon the level of pain the patient feels 1 year after surgery.

Systematic literature reviews on VBRP [11, 12], P4P [13–15], and bundled payment [16, 17] provide mixed evidence of their effect on quality. This is most likely due to the fact that it has proven difficult to summarize and synthesize actual effect on quality due to substantial heterogeneity in the types of outcomes [12]. Further, the link between process measures and patient outcomes are inherently vague and difficult to interpret. Therefore, it has been argued that it is preferable to use distinct outcome measures as a proxy for quality instead of process measures [2, 11, 18, 19]. In particular, patient reported outcome measures (PROM) have gained an important role in the assessment of quality of healthcare [20]. Still, research on the effect of linking reimbursement to PROM is limited [21]. Although VBRP aims to improve quality, there is also some potential pitfalls. For example, it might create incentives for healthcare providers to cherry-pick patients with a more favorable prognosis, which potentially could lead to inequalities in access to healthcare. Studies empirically testing for such effects when introducing a VBRP are scarce, especially within a universal healthcare system since most of published literature has a US setting.

The overall aim of this study was to analyze the effect of a value-based reimbursement program (STHLM-VBRP) on patient reported health outcomes. In addition, we explored whether selection bias towards less complicated patients occurred, regarding medical and socioeconomic factors.

### Healthcare setting

Region Stockholm is one out of 21 regions in Sweden, with the responsibility to provide and finance healthcare, mainly through tax revenues. Hence, the Swedish healthcare system is publicly financed with universal coverage. Both public and private healthcare providers are allowed on the healthcare market. Private healthcare providers must however establish a commissioning contract with each region in which they wish to deliver care. This is done either through the Public Procurement Act or through the Freedom of Choice Act (also known as Patient Choice within healthcare settings). Under the Public Procurement Act, healthcare providers are permitted to a certain volume each year to an individually negotiated price. The Freedom of Choice Act is a more market-inspired contract with no restriction on volume but with a set price, making providers compete based on quality and ultimately the patients’ choice, a requirement for value-based healthcare [19].

Region Stockholm introduced a value-based reimbursement program (STHLM-VBRP) for elective spine surgery at the end of year 2013. Simultaneously, they switched from the Public Procurement Act to The Freedom of Choice Act within elective spine surgery. Elective surgery does not involve any emergency and is therefore scheduled in advance after referral from primary care to the spine surgery specialist. The new reimbursement program covers only private healthcare providers and they performed most of the surgeries, both before and after the introduction of the new reimbursement program. At the time of the introduction, there were three private healthcare providers in Region Stockholm and a fourth provider was accredited in 2017.

### The value-based reimbursement program

The design of the payment affects the efficiency of healthcare providers [12, 22]. When reimbursement programs get complex, the design and interaction of the different payment models get even more essential for understanding consequences. In this section, we therefore explicate the different payment models that constitutes the STHLM-VBRP. In this study we focus on the effect side of the reimbursement program only. Hence, we will not address costs and resource utilization.

Table 1 presents the different categories that are used within the STHLM-VBRP to generate a prospective payment. These categories are based upon diagnostic groups that are used in the national quality registry for spine surgery, Swespine [23].

**Table 1:** Categories used to generate the prospective payment based on diagnosis and surgical procedure in the Stockholm value-based reimbursement program (STHLM-VBRP)

Category	Diagnosis	Surgical procedure
A	Disc herniation	Discectomy
B1	Spinal stenosis	Decompression
B2	Spinal stenosis	Fusion
C	Segmental dysfunction	Fusion
D	Spondylolisthesis	Fusion

**Fig. 1:**
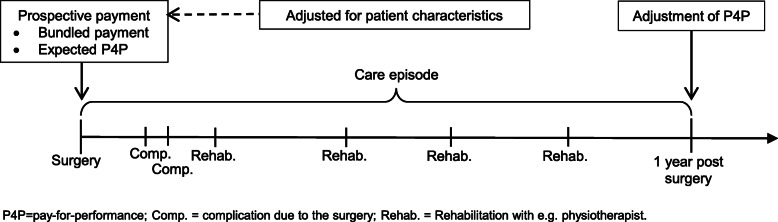
Illustration of the value-based reimbursement program used in elective spine surgery in Region Stockholm (STHLM-VBRP), Sweden. The timeline corresponds to the care episode of 1 year, starting with the surgery. The healthcare provider receives a prospective payment when the surgery is registered. The provider performing the surgery has a cost responsibility for all healthcare utilization related to the spine surgery during the care episode. The prospective payment is adjusted for patient characteristics and includes the bundled payment and the expected performance-based payment (P4P) related to Global Assessment (GA). One year after surgery is the performance-based payment adjusted based on the actual outcome of GA

When the surgical procedure is registered, the healthcare provider receives a prospective payment entailing the bundled payment and the expected performance-based payment (Fig. 1). The bundled payment should cover all healthcare utilization related to the spine surgery (e.g. potential complications, reoperation, rehabilitation visits) during the care episode of 1 year. Thus, the bundled payment extends the cost responsibility to entail healthcare that is provided by other healthcare providers, to stimulate an effective and integrated care chain.

To promote need-based healthcare, differences in financial risk between patients has to be limited. Hence, the prospective payment is adjusted for age, gender, and comorbidity level. Further, procedures that involved surgery on more than two levels of the back generates an additional payment to the provider. Failing to adjust for case-mix leads to an increased risk for “cherry picking”, i.e. providers avoiding clinically complicated patients to the benefit of healthier patients with higher chance of a successful result. Method and results of the calculations of the individual adjustment is presented in Supplementary Material, section A.

To circumvent that healthcare providers stint on necessary care, performance-based payment can be used as a complement to bundle payment. The performance-based payment used in STHLM-VBRP was based on the outcome measure Global Assessment (GA), which is a retrospective transition question asked 1 year after surgery (“How is your back/leg pain today compared to before the surgery?”). The performance-based payment is based on leg pain in category A, B1 and B2, and back pain in category C and D (categories presented in Table 1). The patient could choose between six response options (pain free, much better, somewhat better, unchanged, worse, did not have pain before the surgery) [24]. Data collection was administered and managed by the Swedish quality register for spine surgery (Swespine). Importantly, healthcare providers were not in any way involved in this process.

The expected P4P, which is included in the prospective payment to healthcare providers, is based on national historical outcomes of GA registered in Swespine. One year after surgery, the expected P4P is adjusted according to the actual patient reported outcome of GA. When patients report that the pain has improved more than predicted, the healthcare provider receives an additional payment. When patients report that the pain has improved less than predicted, the healthcare provider has to repay money to Region Stockholm. Hence, the magnitude of the monetary adjustment depends on the discrepancy between the actual and the predicted outcome (based on historical data). Table 2 shows the mean adjustment of the performance-based payment to healthcare providers for different levels of pain, measured with GA, 1 year after surgery. Patients who turned out better than predicted generated a positive adjustment, in the range of 1 to 6% of the prospective payment. Whereas patients that turned out worse than predicted generated a negative adjustment, in the range of − 1 to − 18% of the prospective payment. As Table 2 also shows, there were stronger financial incentives associated with avoiding negative outcomes compared to reaching positive outcomes. More detailed information about the performance-based payment is presented in the Supplementary Material, section B.

**Table 2:** The adjustment of the performance-based payment (P4P) in the Stockholm value-based reimbursement program (STHLM-VBRP)

	The pain is gone	The pain is much better	The pain is slightly better	The pain has not changed	The pain is worse
**Positive adjustment**					
P4P-adjustment	€ 302	€ 92	N/A	N/A	N/A
P4P-adjustment as a share of the prospective payment	6%	1%	N/A	N/A	N/A
**Negative adjustment**					
P4P-adjustment	N/A	€ -44	€ -317	€ -862	€ − 1445
P4P-adjustment as a share of the prospective payment	N/A	-1%	−5%	−12%	−18%

The amounts in the table correspond to the mean adjustment per patient for each pain level in Global Assessment (GA) 1 year after surgery. N/A (not applicable) indicates that there were no patients that generated that adjustment of the performance-based payment (P4P), given their answer on GA

## Methods

### Design and study population

This is a retrospective observational register study, using a before and after design. Patients 18 years or older living in Region Stockholm and subjected to lumbar spine surgery during 2006–2015 were included based on diagnosis (ICD-10) and surgical procedure code (NCSP). The value-based reimbursement program was introduced in October 2013, thus the period contains 7.75 years before the introduction and 2.25 years after the introduction. Data was collected until the end of 2016 to include the one-year follow-up of patients surgically treated in 2015.

### Data sources

Data on diagnosis, surgical procedure, age, gender, total payment (from purchaser to healthcare provider), P4P-adjustment and individual adjustment were extracted from the Stockholm regional patient registry (VAL). Socioeconomic data was extracted from Statistics Sweden. Targeted and non-targeted patient reported outcome measures were extracted from the Swedish spine register (Swespine). The targeted performance measure – global assessment (GA) – is a measure of improvement of clinical symptoms and thus registered solely at the 1-year follow-up. EQ-5D-3L and Oswestry Disability Index (ODI) however, are registered both prior to surgery (baseline) and at 1-year follow-up. Thus, both baseline and 1-year follow up values were extracted for the non-targeted performance measures. EQ-5D-3L is a standardized instrument developed by the EuroQol Group to be used as a measure of health outcome, it comprises five dimensions (mobility, self-care, usual activities, pain/discomfort and anxiety/depression), with three levels (no problems, some problems, and extreme problems). The EQ-5D-3L was converted into a single summary index in Swespine using the tariff by Dolan [25]. This index value can vary from − 0.52 to 1 and facilitates the calculation of quality-adjusted life years (QALYs) [26]. The ODI is one of the most commonly recommended condition specific outcome measure for spinal disorders [27, 28]. The ODI comprises ten items; pain intensity, personal care (washing, dressing, etc.), lifting, walking, sitting, standing, sleeping, sex life, social life, and traveling. For each item there are six severity levels scoring from 0 to 5. The total possible score is 50 and a standardized formula is used to transform the score to a percentage score of disability, where 0% corresponds to no disability and 100% corresponds to full disability.

The National Board of Health and Welfare anonymized and interlinked data from the patient registers, Swespine and Statistics Sweden. Data was obtained with ethical approval (Dnr 2015/94–31) from the Regional Ethical Review Board in Linköping, Sweden.

Monetary values have been adjusted to the 2016 price level and presented in EUR with an exchange rate corresponding to 1 SEK = 0.11 EUR.

### Analysis

To analyze the effect of the STHLM-VBRP on patient reported outcome measures we compared the distribution of answers on GA before and after the introduction of the reimbursement program. Global assessment is the targeted outcome measure in STHLM-VBRP but only measured after the surgery. Therefore we chose to analyze the change in EQ-5D-3L and ODI that is registered both before and after the surgery. It also made it possible to analyze whether there was any difference between targeted and non-targeted PROMs. To make sure that any potential effect on PROM was not caused by selection bias we compared the case-mix of patients surgically treated before and after the introduction of the STHLM-VBRP.

To analyze how the reimbursement program affected GA, we performed a chi-square test. Patients that had answered that they had no pain before the surgery were excluded from the analysis.

To analyze the association between the STHLM-VBRP and non-targeted outcome measures (EQ-5D-3L and ODI) we used segmented regression analysis to assess potential changes in level and trend over time [29]. We controlled for baseline level and trend using Model 1 to estimate changes in level and trend associated with the introduction of STHLM-VBRP. The introduction of STHLM-VBRP interrupts the time series and creates two segments of interest. The following regression model was specified to estimate the monthly level and trend of EQ-5D-3L and ODI score, at baseline, at 1-year follow-up, and the change after surgery (i.e. the difference between 1-year follow-up and baseline level).

Model 1 *Y*_*t*_ = *β*_0_ + *β*_1_ ∗ *time*_*t*_ + *β*_2_ ∗ *VBRP*_*t*_ + *β*_3_ ∗ *time after VBRP*_*t*_ + *β*_4_ ∗ *July*.

The dependent variable *Y*_*t*_ in month *t* (i.e. EQ-5D-3L level or ODI score) was explained by four independent variables where *β*_0_ estimated the baseline level at time zero. The variable *time* indicated time in months at time *t* from the start of the observation period to the end (2006–2016) where *β*_1_ estimated the monthly change (i.e. the baseline trend). The dichotomous variable *VBRP* indicated whether time *t* occurred before (VBRP = 0) or after (VBRP = 1) the introduction of STHLM-VBRP, corresponding to month 92 in the time series. The *β*_2_-coefficient estimates the change in the outcome level after the introduction of STHLM-VBRP. The variable *time after VBRP* indicates the number of months after the introduction of STHLM-VBRP, coded 0 before STHLM-VBRP and (time-91) after the introduction of STHLM-VBRP, the *β*_3_-coefficient estimates the change in the baseline trend after the introduction of STHLM-VBRP. The time coefficient *β*_1_ is present through the entire time period, 2006–2016. Consequently, the sum of *β*_1_ and *β*_3_ is the post-intervention slope. The variable *July* is a dummy variable (0 or 1 to indicate the month of July), *β*_4_ estimates the impact the month of July has on the outcome (due to summer holidays far less patients undergo surgery during this month).

To analyze how the introduction of the STHLM-VBRP in relation to medical and socioeconomic factors affected the odds of a successful surgery, we performed a logistic regression analysis presented in Model 2. For a surgery to be successful the patient had to answer “the pain is gone”, “the pain is much better” or “the pain is slightly better” on GA. Patients that had answered “had no pain before the surgery” were excluded from the analysis since that option cannot be put on an ordinal scale. We used Charlson comorbidity index [30] to calculate comorbidity level based on diagnoses registered in the Stockholm regional patient registry.

*Model 2* Successful surgery = β_0_ + β_1_ ∗ *VBRP* + β_2_ ∗ *age* + β_3_ ∗ female gender + β_4_ ∗ comorbidity level + β_5_ ∗ low educational level + β_6_ ∗ *income* + β_7_ ∗ *born outside of Europe*

We controlled for case-mix by using the logistic regression specified in Model 3. The odds of being surgically treated after the introduction of VBRP is compared to being surgically treated before the introduction of VBRP, with regards to age, gender, comorbidity level, educational level, income level and place of birth. Using the same variables as in Model 2 allowed us to analyze whether patient characteristics with lower odds of a successful surgery also had lower odds of being surgically treated.

*Model 3* Surgically treated after the introduction of VBRP = β_0_ + β_1_ ∗ *age* + β_2_ ∗ female gender + β_3_ ∗ comorbidity level + β_4_ ∗ low educational level + β_5_ ∗ *income* + β_6_ ∗ *born outside of Europe*

Patients with missing values in reimbursement were excluded from the analysis. Statistical significance was assessed at the 5% level. Analyses were performed using SAS 9.4.

## Result

In Region Stockholm, 10,389 patients were surgically treated for low back pain between 2006 and 2015. Out of them, 6738 patients were treated before the introduction of VBRP and 3651 after the introduction. Baseline characteristics of surgically treated patients before and after the introduction of the VBRP is presented in Table 3. The comorbidity level increased from an average of 0.24 to 0.31. Further, the proportion of patients with at least one registered comorbidity increased from 15% to 19%. The ODI level however, decreased with 0.7 percentage points, indicating a less impaired population. The mean annual income increased among patients surgically treated after the introduction from €27,449 to €31,185. The proportion of patients being employed increased from 53% to 55% and patients born outside of Europe increased from 8% to 12%.

**Table 3:** Baseline characteristics of surgically treated patients before and after the introduction of the Stockholm value-based reimbursement program (STHLM-VBRP)

Variable	Mean (SD)		Δ	t-test (p)	Wilcoxon (p)
	Without VRBP ***n*** = 6738	With VBRP ***n*** = 3651			
Age	56.49 (15.34)	56.45 (15.77)	0.036	0.910	
Female (%)	53.77 (49.86)	52.12 (49.96)	1.65	0.108	0.385
BMI	26.65 (7.10)	26.65 (7.94)	−0.0009	0.995	
Comorbidity level (CCI)	0.24 (0.705)	0.31 (0.78)	−0.0652	<.0001	
At least one comorbidity (%)	15 (35.61)	19 (39.22)	−4	<.0001	0.007
EQ-5D prior to surgery	0.377 (0.325)	0.364 (0.330)	0.013	0.061	0.273
ODI prior to surgery	41.88 (15.87)	41.16 (16.409)	0.722	0.041	0.164
Annual income (€)	27,449 (26053)	31,185 (44929)	33,915	<.0001	
Low educational level (%)	20.48 (40.36)	20.05 (40.04)	0.432	0.602	0.943
Employed (%)	52.67 (49.93)	54.73 (49.78)	2.06	0.045	0.097
Born outside of Europe (%)	8.22 (27.47)	12.01 (29.34)	3.79	<.0001	<.0001

*Note*: *SD* Standard deviation, *BMI* Body Mass Index (measured as weight/height^2^), *CCI* Charlson Comorbidity Index, Low educational level refers to patients that have not finished secondary education

### The targeted performance measure - GA

**Fig. 2:**
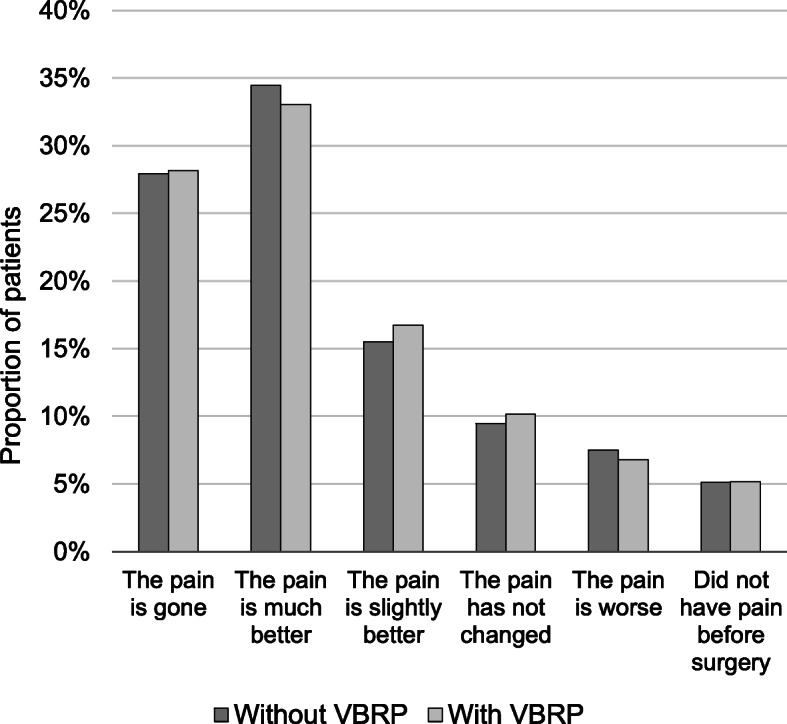
Patients’ answer on Global Assessment before and after the introduction of the value-based reimbursement program

Both before and after the introduction of VBRP, 71% of the patients answered GA. There was no difference in the distribution of the patients’ answer on GA (χ^2^ (4, *N* = 6964) = 4.64, *p* = 0.326). Thus, linking the performance-based payment to GA did not change the pain patients experienced 1 year after surgery. The distribution of answers is illustrated in Fig. 2. The fraction of patients that experienced a successful surgery (i.e. *the pain is gone*, *much better*, or *slightly better*) corresponded to 78%, both before and after the introduction of the new reimbursement program. Further, the fraction of patients that did not have pain before the surgery remained at 5% after the introduction.

### The non-targeted performance measures, EQ-5D-3L and ODI

**Fig. 3:**
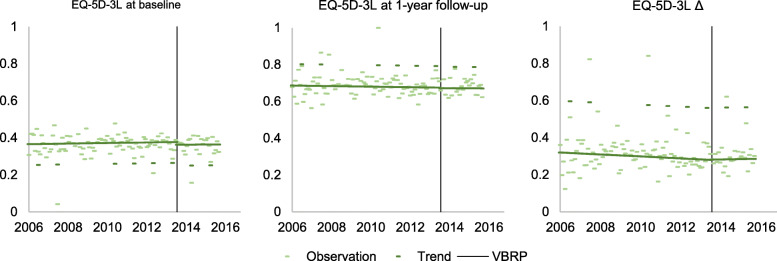
The mean monthly EQ-5D-3L level of surgically treated patients. The mean monthly EQ-5D-3L level at baseline, 1-year follow up and the difference between follow-up and baseline (Δ-score, i.e. the change after surgery) among patients surgically treated 2006–2015. The vertical line indicates the introduction of the STHLM-VBRP at the end of 2013

Table 4 presents the estimates for level and trend in EQ-5D-3L prior to surgery (baseline), at 1-year follow-up and change after surgery (the difference between follow-up and baseline, Δ-score) before and after the introduction of STHLM-VBRP. An illustration of the average level of EQ-5D-3L for patients surgically treated between 2006 and 2015 is illustrated in Fig. 3. Patients surgically treated in 2006, had an EQ-5D-3L level of 0.365 prior to surgery (*p*-value <.0001). There was no month-to-month change in EQ-5D-3L, neither before or after the introduction of STHLM-VBRP (*p*-values 0.488 and 0.956 respectively), nor the level was affected (p-value 0.483). The 1-year follow-up level of EQ-5D-3L of patients surgically treated in 2006 corresponded to 0.686 (p-value <.0001). There was no change in trend nor level before and after the introduction of STHLM-VBRP. The change (Δ) in health after surgery was 0.319 among patients surgically treated in 2006 (*p*-value <.0001). As illustrated in Fig. 3, there were no changes in trend of level after the introduction of SHTLM-VBRP, neither prior to surgery (baseline), at 1-year follow-up or in improvement (the difference between follow-up and baseline). Thus, the value-based reimbursement program had no effect on level or trend of health related quality of life measured with EQ-5D-3L.

**Table 4:** Parameter estimates predicting the mean monthly EQ-5D-3L level among surgically treated patients

Parameter	EQ-5D-3L baseline			EQ-5D-3L 1-year follow up			EQ-5D-3L Δ (follow up-baseline)		
	Estimate	SE	***p***-value	Estimate	SE	***p***-value	Estimate	SE	***p***-value
**Intercept**	0.365	0.01	<.0001	0.686	0.011	<.0001	0.319	0.016	<.0001
**Time**	0.0001	0	0.488	−0.0001	0.0002	0.532	0.0004	0.0003	0.143
**VBRP**	−0.015	0.021	0.483	−0.004	0.022	0.85	0.001	0.033	0.972
**Time after**	−0.0001	0.001	0.956	0.0001	0.001	0.938	0.001	0.002	0.751
**July**	−0.113	0.018	<.0001	0.117	0.018	<.0001	0.279	0.027	<.0001

*Note*: *SE* Standard error, *Intercept,* the EQ-5D-3L level in January 2006; *Time,* number of months from January 2006; *VBRP,* indicates the introduction of the STHLM-VBRP in the end of 2013; which is 92 months after January 2006 (Time = 92); *Time after, number of months* after the introduction of VBRP (hence Time-91); *July,* indicates the month of July

Parameter estimates from the segmented regression analysis predicting the mean monthly EQ-5D-3L level among surgically treated patients before and after the introduction of the STHLM-VBRP, 2006–2015. The introduction of the reimbursement program had no effect on level (VBRP) nor trend (Time after) of EQ-5D-3L

**Fig. 4:**
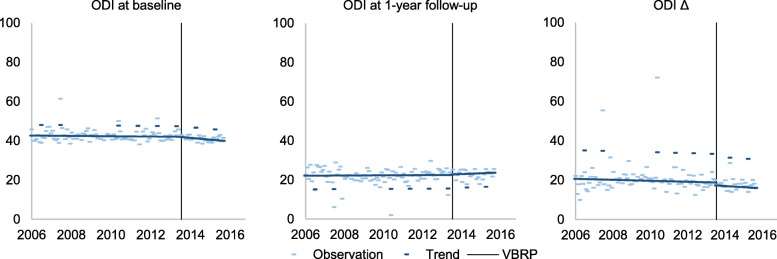
The mean monthly ODI level of surgically treated patients. The mean monthly ODI level at baseline, 1-year follow up and the difference between follow-up and baseline (Δ-score, i.e. the change after surgery) among patients surgically treated 2006–2015. The vertical line indicates the introduction of the STHLM-VBRP at the end of 2013

Table 5 presents the estimates for level and trend in ODI prior to surgery (baseline), at 1-year follow-up and the change after surgery (the difference between follow-up and baseline, Δ-score) before and after the introduction of STHLM-VBRP. An illustration of the average value of ODI for patients surgically treated between 2006 and 2015 is illustrated in Fig. 4. The disability level prior to surgery among patients surgically treated in 2006 was 42.68%. Neither level nor trend in ODI was affected by the introduction of the new reimbursement program. The disability level at 1-year follow up among patients surgically treated in 2006 was 22.14% and there was no change in level or trend at the introduction of the STHLM-VBRP. The relative improvement (Δ) in disability level among patients surgically treated in 2006 corresponded to a 20.61 percentage point decrease. The introduction of STHLM-VBRP had no effect on level nor trend of patients’ disability level measured with ODI.

**Table 5:** Parameter estimates predicting the mean monthly ODI level among surgically treated patients

Parameter	ODI baseline			ODI 1-year follow up			Δ ODI (follow up-baseline)		
	Estimate	SE	***p***-value	Estimate	SE	***p***-value	Estimate	SE	***p***-value
**Intercept**	42.68	0.54	<.0001	22.140	0.74	<.0001	20.61	1.21	<.0001
**Time**	−0.008	0.01	0.451	0.004	0.01	0.776	−0.02	0.02	0.372
**VBRP**	−0.069	1.14	0.952	0.250	1.57	0.875	−1.41	2.54	0.579
**Time after**	−0.066	0.06	0.309	0.030	0.09	0.749	−0.03	0.14	0.819
**July**	5.460	0.94	<.0001	−6.990	1.28	<.0001	14.57	2.09	<.0001

*Note*: *SE* Standard Error, *Intercept,* The ODI level in January 2006; *Time,* number of months from January 2006; *VBRP,* indicates the introduction of the STHLM-VBRP at the end of 2013; which is 92 months after January 2006 (Time = 92); *Time after,* number of months after the introduction of VBRP (Time-91); *July,* indication of the month of July

Parameter estimates from the segmented regression analysis predicting the mean monthly Oswestry disability index (ODI) level among surgically treated patients before and after the introduction of the STHLM-VBRP, 2006–2015. The introduction of the reimbursement program had no effect on level (VBRP) nor trend (Time after) of ODI

### Case-mix

The odds ratio of a successful surgery is presented in Table 6 (based on Model 2). Age (OR = 0.96; CI 0.96 to 0.97), low educational level (OR = 0.79; CI 0.69 to 0.91) and born outside of Europe (OR = 0.56; CI 0.45 to 0.69) was associated with lower odds of a successful surgery. Thus, socioeconomic factors seem to affect the chance of a successful surgery.

**Table 6:** Odds ratio (OR) estimates to experience a successful surgery, 2006–2015

Variable	Point Estimate	95% Confidence Limits		*p*-value
	1.075	0.950	1.216	0.2521
Age	0.963	0.959	0.967	<.0001
Female	1.020	0.904	1.150	0.7529
Comorbidity level (CCI)	0.957	0.887	1.031	0.2463
Low educational level	0.791	0.688	0.910	0.001
Annual income	1	1	1	<.0001
Born outside of Europe	0.555	0.448	0.689	<.0001

*Note*: *STHLM-VBRP* Stockholm value-based reimbursement program, *CCI* Charlson comorbidity index, Low educational level refers to patients that have not finished secondary education

Odds ratio estimates to experience a successful surgery with respect to the introduction of the STHLM-VBRP and patient characteristics. Odds ratios above 1.0 indicate a higher odds of a successful surgery in that category than in the reference group, whereas odds ratios below 1.0 indicates a lower odds of a successful surgery

Table 2 showed that patients with risk factors such as comorbidity level, low educational level and born outside of Europe increased after the introduction of the STHLM-VBRP. It also showed that the income level increased, which could be an indication of cherry-picking. The odds of being surgically treated within the STHLM-VBRP compared to before the introduction is presented in Table 7. The odds of being surgically treated was higher among patients with a high comorbidity level after the introduction of the VBRP (OR = 1.13; CI 1.07–1.20). This was also the case for patients that were born outside of Europe (OR = 1.57; CI 1.39–1.83). However, the income level did not affect the odds of being surgically treated (OR = 1; CI 1–1).

**Table 7:** Odds ratio estimates for being surgically treated after the introduction of the Stockholm value-based reimbursement program (STHLM-VBRP)

Effect	Point Estimate	95% Confidence Limits		*p*-value
Age	1	0.997	1.003	0.9302
Female	0.992	0.911	1.080	0.8535
Comorbidity level (CCI)	1.133	1.069	1.201	<.0001
Low educational level	0.977	0.879	1.085	0.6601
Annual income	1	1	1	<.0001
Born outside of Europe	1.596	1.388	1.834	<.0001

*Note*: *CCI* Charlson comorbidity index, Low educational level refers to patients that have not finished secondary education

Odds ratio estimates for being surgically treated after the introduction of the value-based reimbursement program as regards to patient characteristics. Odds ratios above 1.0 indicate a higher odds of being surgically treated after the introduction of the value-based reimbursement program in that category than in the reference group, whereas odds ratios below 1.0 indicates a lower odds of being surgically treated

## Discussion

In this study we analyzed the effect of a value-based reimbursement program (STHLM-VBRP) on patient reported outcome measures (PROM). Our results clearly show that the introduction of STHLM-VBRP had no effect on any of the PROMs included in the study (GA, EQ-5D-3L and ODI). The level of EQ-5D-3L and ODI prior to surgery and at follow-up are similar to the level in other published studies [31–33], indicating that the population is similar to other contexts. Thus, we found no indication of P4P distorting the focus from non-targeted PROMs. The lack of effect on targeted or non-targeted outcome measures is in line with previously published results [12, 13, 21, 34, 35]. Nonetheless, it is important to discuss the lack of effect and how this relates to the incentive structure imposed by the reimbursement program [11, 36]. A performance-based payment can serve as a compliment to a bundled payment to prevent healthcare providers from stinting on necessary care. In the case of the STHLM-VBRP the providers, however, only observed the adjustment part of the performance-based payment. Thus, the full P4P was not observed by the healthcare provides which might contribute to the fact that it had no overall effect. It should also be noted that the financial incentive of the P4P within the STHLM-VBRP was primarily focused on avoiding negative outcomes rather than incentivizing positive outcomes. Thus, the financial incentives associated with the P4P within the STHLM-VBRP was more of a whip than a carrot for the healthcare providers. This incentive structure makes it even more important for healthcare providers to come to an understanding with which patients that actually benefit from a surgery and which patients that do not. Something which is continuously debated within spine surgery.

Failing to adjust the reimbursement for variation in risk factors among patient may cause providers to attempt shifting their case-mix of patients toward patients with higher probability of positive outcomes, i.e. cherry-picking [9, 21, 28, 37]. This has been considered to be the largest challenge facing bundled payments in spine surgery [38]. Our results do not indicate any shift towards a healthier case-mix, rather the contrary. The number of patients with risk-factors such as comorbidities, low educational level and born outside of Europe increased after the introduction of the VBRP. Hence, the value-based reimbursement program did not encourage discrimination against sicker patients. However, the income was higher among patients surgically treated after the introduction of the value-based reimbursement. This could be an indication that a VBRP contributes to increased inequalities in access to healthcare. However, future studies need to further explore such potential effects and whether they could be reliably linked to the reimbursement program.

Some limitations of our study should be noted. First, our dataset did not include patients referred to a specialist that were not surgically treated. Hence, we cannot rule out that cherry-picking or shift in indications occurred in that part of the care chain. The indications for surgery within elective spine surgery are sometimes vague and highly debated. Some surgical procedures only have a modestly better effect but are more costly and carries a greater risk of adverse events than non-surgical management [6, 39]. Vague indications might lead to an increased procedural volume of spine surgery without regard to quality, thus drive cost and diminish the value of spine care [40, 41]. Potentially can VBRPs weed out providers delivering high quantity/low value care and ultimately reward those who are delivering superior outcomes [28]. The number of surgically treated patients increased with STHLM-VBRP without any effect on PROM. A potential explanation is the removal of the volume restriction that private healthcare providers were facing before STHLM-VBRP, meaning that the increase was caused by a previously unmet demand. However, costs and resource utilization must be investigated to assess whether the STHLM-VBRP increased the value or not.

Second, our data material only covers the first 2 years with the new reimbursement program. Previous research by Song et al. [42] have showed that larger improvements in quality is likely to occur during the second year when implementing a VBRP. Thus, it takes time for providers to adopt to the structures of a new reimbursement program [43], which can be a possible explanation to the lack of noticeable effects on patient reported outcome measures [44]. In our material, there was an increase in volume during the third year (in 2016), but we had no data to assess the patient reported outcome measures during this period. Further it is common with transition periods [35] that is characterized with so called “child diseases” that occur during the implementation and may cause a drop in quality of care [4]. Thus, in further studies with a longer timeframe it would be plausible to use a “wash-out” period to remove potential transition effects. Nevertheless, this limitation is simultaneously a strength since it reflects the reality providers were facing during the first 2 years of using a VBRP. Due to the observational approach with a natural experiment design of our study we can only test for association and not causality, thus our analysis relies on pre-post comparisons without a comparison group that was not exposed to the intervention. To adjust for this we used segmented regression analysis to assess whether there had been any notable external changes in trend or level.

## Conclusions

We found no effect, neither positive nor negative, when studying the effect of the value-based reimbursement program on patient reported outcome measures. However, we found an increased share of surgically treated patients with risk factors such as having comorbidities and being born outside of Europe after the introduction of the program. Hence, the value-based reimbursement program did not encourage discrimination against sicker patients. However, patients that were surgically treated after the introduction had a higher income. This indicates that a VBRP may contribute to increased inequalities in access to healthcare. Future research is needed to study the effect on resource utilization and costs, but also how a value-based reimbursement program affects inequalities in access to healthcare.

## Supplementary information

**Additional file 1: Section A.** The individual adjustment **Section B.** The performance-based payment.

## Data Availability

The data that support the findings of this study are available from Region Stockholm and Statistics Sweden but restrictions apply to the availability of these data, which were used under license for the current study, and so are not publicly available. Data are however available from the authors upon reasonable request and with permission of Region Stockholm, Statistics Sweden and the Regional Ethical Review Board in Linköping, Sweden.

## Abbreviations

DRG: Diagnosis-Related Group
GA: Global Assessment
EQ-5D-3L: EuroQol group 5 dimensions 3 levels
ODI: Oswestry Disability Index
P4P: Pay for Performance
PROM: Patient Reported Outcome Measure
QALY: Quality Adjusted Life Years
STHLM-VBRP: Stockholm Value-Based Reimbursement Program
VBRP: Value-Based Reimbursement Program
