# Correction to: A pain relieving reimbursement program? Effects of a value-based reimbursement program on patient reported outcome measures

**DOI:** 10.1186/s12913-020-05714-4

**Published:** 2020-09-17

**Authors:** Thérèse Eriksson, Hans Tropp, Ann-Britt Wiréhn, Lars-Åke Levin

**Affiliations:** 1 Department of Health, Medicine and Caring Sciences (HMV), Centre for Medical Technology Assessment (CMT), Linköping University, SE-581 83 Linköping, Sweden; 2 Department of Biomedical and Clinical Sciences, Linköping University, SE-581 83 Linköping, Sweden; 3 Research and Development Unit in Region Östergötland and Department of Medical and Health Sciences, Linköping University, SE-581 83 Linköping, Sweden

**Correction to: BMC Health Serv Res (2020) 20:805**

**https://doi.org/10.1186/s12913-020-05578-8**

Following publication of the original article [1], the authors identified some errors in Table 6. The correct table and caption have been included in this correction, and the original article has been corrected.

**Table 6:** Odds ratio (OR) estimates to experience a successful surgery, 2006–2015

	Point Estimate	95% Confidence Limits		***p***-value
**STHLM-VBRP**	1.075	0.950	1.216	0.2521
**Age**	0.963	0.959	0.967	<.0001
**Female**	1.020	0.904	1.150	0.7529
**Comorbidity level (CCI)**	0.957	0.887	1.031	0.2463
**Low educational level**	0.791	0.688	0.910	0.001
**Annual income**	1	1	1	<.0001
**Born outside of Europe**	0.555	0.448	0.689	<.0001

Note: *STHLM-VBRP* Stockholm value-based reimbursement program, *CCI* Charlson comorbidity index, Low educational level refers to patients that have not finished secondary education

Table 6 legend: Odds ratio estimates to experience a successful surgery with respect to the introduction of the STHLM-VBRP and patient characteristics. Odds ratios above 1.0 indicate a higher odds of a successful surgery in that category than in the reference group, whereas odds ratios below 1.0 indicates a lower odds of a successful surgery
