# Supplementary material for: A pain relieving reimbursement program? Effects of a value-based reimbursement program on patient reported outcome measures
Source: BMC Health Serv Res. 2020 Aug 27;20:805. doi: 10.1186/s12913-020-05578-8 (PMC7450562; doi:10.1186/s12913-020-05578-8)
Supplement: Supplementary file 1 — Additional file 1: Section A. The individual adjustment Section B. The performance-based payment. [file 12913_2020_5578_MOESM1_ESM.pdf]

Supplementary material

Contents

Section A: The individual adjustment 1
Method 1
Result 2
Table 7 The individual adjustment of the prospective payment in STHLM-VBRP. 2
Table 8 Factors affecting the outcome of the individual adjustment 3
Section B: The performance-based payment 3
Method 3
Result 3
Table 9 The outcome of the P4P-adjustment 4
Fig. 5 The range of the performance-based adjustment. 4
Table 10 The range of the adjustment of the P4P-payment and the range of the P4P-payment that was listed in the commissioning contract, in relation to the outcome of global assessment (GA) and category. 5

Section A: The individual adjustment

Method

The prospective payment was adjusted based on patient characteristics to promote a need-based healthcare and avoid cherry-picking. We used descriptive statistics to describe the outcome of the individual adjustment in the Stockholm value-based reimbursement program (STHLM-VBRP), aggregated from patient level data. We used regression analysis to assess the effect of gender, age and comorbidity level on the individual adjustment.

Result

The prospective payment was adjusted for 84 percent of the patients during the first three years of the STHLM-VBRP. On average, patients within category C (segmental dysfunction+fusion) generated the most positive adjustment (i.e. the highest pay-out to the provider from the purchaser), with a mean of € 219 and a min-max of € -158 and € 1,077 respectively (Table 7).

Table 7 The individual adjustment of the prospective payment in STHLM-VBRP. The proportion of patients that generated an adjustment of the prospective payment and the proportion of with a positive adjustment (i.e. the patient has higher expected costs compared to the "standard" patient, thus the health care provider receives a larger payment). Further, the table also contains the mean of positive and negative adjustments, minimum and maximum payment and the minimum and maximum of the adjustment as a share of the payment.

	N	n/N	Share with positive adjustment	Mean positive adj. € (SD)	Mean negative adj. € (SD)	Min €	Max €	Min share	Max share
**A**	951	88%	31%	65 (85)	-19 (8)	-26	481	-1%	17%
**B1**	1793	87%	51%	182 (132)	-180 (132)	-640	734	-12%	16%
**B2**	261	88%	55%	163 (122)	-109 (66)	-237	563	-2%	7%
**C**	462	86%	49%	219 (213)	-56 (41)	-158	1,077	-2%	9%
**D**	184	84%	52%	114 (134)	-80 (63)	-209	642	-2%	6%

Note: A=Disc herniation+discectomy, B1=Spinal stenosis+decompression, B2=Spinal stenosis+fusion, C=Segmental dysfunction+fusion, D=Spondylolisthesis+fusion, N=number of patients within each category, n/N=the share of patients that generated an adjustment of the prospective payment

The most negative adjustment mean (i.e. the highest amount the provider had to repay the purchaser) was found in category B1, amounting to an average of € -180 with a minimum of € -640 and a maximum of € 734. Category B1 also had the widest range of the individual adjustment as a share of the bundled payment, ranging from -12% to 16%. Age, female gender and comorbidity level had a positive effect on the size of the individual adjustment of the prospective payment (Table 8). Thus, the individual adjustment seems to control for differences in case-mix since the healthcare provider receives a higher payment depending on age, gender and comorbidity level.

Table 8 Factors affecting the outcome of the individual adjustment. Association between patient characteristics and the size of the individual adjustment. It is stated in the contract between health care providers and Region Stockholm, that the prospective payment should be adjusted based on age, gender and comorbidity level.

Parameter	Estimate	Standard Error	t Value	Pr > |t|
**Intercept**	-2690.55	89.27729	-30.14	<.0001
**Age**	46.3986	1.555531	29.83	<.0001
**Female**	323.325	47.66485	6.78	<.0001
**Comorbidity (CCI)**	159.5333	31.12632	5.13	<.0001

Note: CCI=Charlson comorbidity index

Section B: The performance-based payment

Method

The size of the P4P-adjustment was linked to the answer on GA and put in relation to what was listed in the contract between purchaser and provider, i.e. the health care providers' expectations given the GA result and patient characteristics. Further, we performed a logistic regression (Model 3) analysis to test whether VBRP or case-mix affected the odds ratio of a successful surgery. For a surgery to be successful the patient had to answer "the pain is gone", "the pain is much better" or "the pain is slightly better" on GA. Patients that had answered "had no pain before the surgery" were excluded from the analysis.

Result

On average, 32 percent of the patients generated a P4P-adjustment to the provider of which 61 percent were positive, i.e. the provider received an additional payment from the purchaser. Category D had the highest share of positive P4P-adjustments (78 percent) and category A had the lowest share (49 percent). The size of the adjustment of the P4P-payment was on average ± 8 percent of the prospective payment. Including patients that did not generate an adjustment however, the P4P-share of the prospective payment was ±3 percent. The largest positive mean adjustment was found in category B2, amounting to € 673 with a minimum of € -2,260 and a maximum of €1,783 (Table 9). Category D had the largest negative mean adjustment corresponding to € -1,008 with a minimum of € -2,281 and a maximum of € 1,285. Category B1 had the widest range of the P4P-adjustment in relation to the prospective payment, ranging from -20 to 20 percent.

Table 9 The outcome of the P4P-adjustment. The first column presents the proportion of patients that generated a P4P-adjustment. In the second column it is the proportion that generated a positive adjustment, followed by the mean of positive (i.e. payment from purchaser to provider) and negative (i.e. payment from provider to purchaser) adjustments, minimum and maximum adjustment in absolute and relative to the prospective payment.

	n/N	Share of patients with positive P4P-payment	Mean positive P4P € (SD)	Mean negative P4P € (SD)	Min €	Max €	Minimum share of bundled payment	Maximum share of bundled payment
**A**	32%	49%	229 (83)	-212 (248)	-955	358	-23%	12%
**B1**	31%	58%	445 (251)	-562 (378)	-1,327	1,309	-20%	20%
**B2**	37%	60%	673 (415)	-964 (660)	-2,260	1,783	-18%	20%
**C**	30%	60%	484 (393)	-966 (618)	-2,185	1,707	-18%	19%
**D**	29%	78%	572 (410)	-1,008 (727)	-2,281	1,285	-18%	14%

Note: A=Disc herniation+discectomy, B1=Spinal stenosis+decompression, B2=Spinal stenosis+fusion, C=Segmental dysfunction+fusion, D=Spondylolisthesis+fusion. P4P=pay-for-performance.

The distribution of the P4P-adjustment was within the range listed in the STHLM-VBRP contract for all patient categories (Figure 5), except from category A where the actual range had a lower minimum than listed.

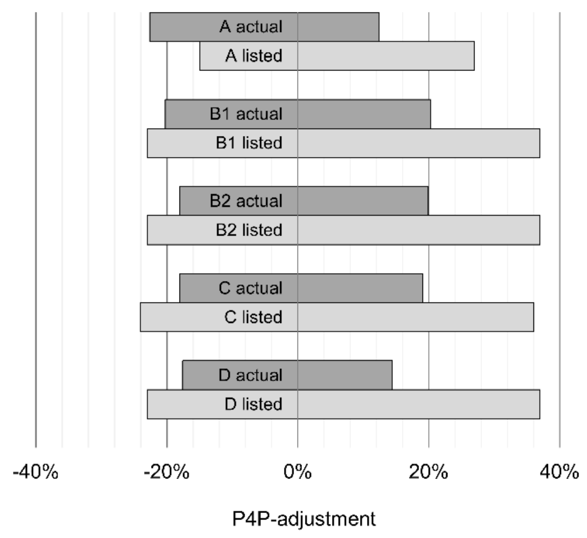

Note: A=Disc herniation+discectomy, B1=Spinal stenosis+decompression, B2=Spinal stenosis+fusion, C=Segmental dysfunction+fusion, D=Spondylolisthesis+fusion.

Fig. 5 The range of the performance-based adjustment. The adjustment of the performance based payment in comparison to the range listed in the STHLM-VBRP contract. The x-axis show the P4P-adjustment as a share of the prospective payment.

In the case of category A, the health care providers had to pay back a larger amount of money than listed in the reimbursement contract (Table 10). Regarding the P4P-adjustment in relation to the answers on GA, the health care providers either got a lower payment or had to pay back a larger amount of money than listed in the STHLM-VBRP contract. Thus, the health care providers got less payed than listed in the contract for patients whose pain improved and they had to pay back more than listed for patients whose pain got worse.

Table 10 The range of the adjustment of the P4P-payment and the range of the P4P-payment that was listed in the commissioning contract, in relation to the outcome of global assessment (GA) and category.

Outcome GA	P4P	A	B1	B2	C	D
**Gone**	Actual	0 to 12	0 to 20	0 to 20	0 to 19	0 to 14
	Listed	13 to 27	17 to 37	17 to 37	16 to 36	17 to 37
**Much better**	Actual	-2 to 2	0 to 8	0 to 12	0 to 6	0 to 5
	Listed	6 to 20	7 to 27	7 to 27	6 to 26	7 to 27
**Slightly better**	Actual	-10 to 0	-8 to 0	-7 to 0	-6 to 0	-6 to 0
	Listed	-1 to 13	-3 to 17	-3 to 17	-4 to 16	-3 to 17
**Unchanged**	Actual	-17 to 0	-14 to 0	-13 to 0	-13 to 0	-10 to 0
	Listed	-8 to 6	-13 to 7	-13 to 7	-14 to 6	-13 to 7
**Worse**	Actual	-23 to 0	-20 to 0	-18 to 0	-18 to 0	-18 to 0
	Listed	-15 to -1	-23 to -3	-23 to -3	-24 to -4	-23 to -3
**Did not have pain before surgery**	Actual	0	0	0	0	0
	Listed	0	0	0	0	0

Note: A=Disc herniation+discectomy, B1=Spinal stenosis+decompression, B2=Spinal stenosis+fusion, C=Segmental dysfunction+fusion, D=Spondylolisthesis+fusion. GA=Global assessment. Actual=The perceived adjustment of the P4P-payment one year after surgery that followed if the outcome of GA deviated from the expected. Listed=the adjustment the healthcare provider was facing, whereas listed was the range of adjustment stated in the commissioning contract.
